# Artificial intelligence and multi agent based distributed ledger system for better privacy and security of electronic healthcare records

**DOI:** 10.7717/peerj-cs.323

**Published:** 2020-11-30

**Authors:** Fahad F. Alruwaili

**Affiliations:** Computer Science, College of Computing and Information Technology, Shaqra University, Shaqra, Kingdom of Saudi Arabia

**Keywords:** Artificial intelligence, Agents and multi-agent systems, Adaptive & self-organizing systems, Electronic health records, Distributed ledger systems

## Abstract

**Background:**

Application of Artificial Intelligence (AI) and the use of agent-based systems in the healthcare system have attracted various researchers to improve the efficiency and utility in the Electronic Health Records (EHR). Nowadays, one of the most important and creative developments is the integration of AI and Blockchain that is, Distributed Ledger Technology (DLT) to enable better and decentralized governance. Privacy and security is a critical piece in EHR implementation and/or adoption. Health records are updated every time a patient visits a doctor as they contain important information about the health and wellbeing of the patient and describes the history of care received during the past and to date. Therefore, such records are critical to research, hospitals, emergency rooms, healthcare laboratories, and even health insurance providers.

**Methods:**

In this article, a platform employing the AI and the use of multi-agent based systems along with the DLT technology for privacy preservation is proposed. The emphasis of security and privacy is highlighted during the process of collecting, managing and distributing EHR data.

**Results:**

This article aims to ensure privacy, integrity and security metrics of the electronic health records are met when such copies are not only immutable but also distributed. The findings of this work will help guide the development of further techniques using the combination of AI and multi-agent based systems backed by DLT technology for secure and effective handling EHR data. This proposed architecture uses various AI-based intelligent based agents and blockchain for providing privacy and security in EHR. Future enhancement in this work can be the addition of the biometric based systems for improved security.

## Introduction

The rapid improvement of digitizing the healthcare has led to the creation of huge electronic records of patients. Such progress paves a way for unparalleled demands for the protection of healthcare data and at the time of utilizing and transferring these data. E-Health systems can be a better alternative for maintaining the medical records globally and connectedly and can be further accessed the clinical information on the basis of its requirement (Wiljer et al., 2008). There is a rapid increase in the applicants of EHR in E-Health which uses the mobile based devices in order to provide medical assistance. Some of the medical services such as acquisition of data through online, and also in person, transferring these data towards other medical service providers etc. The EHR is a digital based medical data preserving and processing platform which is easily accessible to the patient as well as the doctors (Zurita & Nøhr, 2004). The main aim of this EHR is to monitor and to maintain the patients’ medical data more securely. This includes the overall medical history of the patient, current health condition, demographic details about the patient etc. This EHR acts as a repository for storing, transferring the medical data more securely (Kumar & Lee, 2011). The patient, doctor and the medical service provider can fetch the data whenever and where ever necessary. Service providers need to update the given services to maintain consistency. In fact, various regulations and standards have been proposed by earlier researchers (OECD, 2013) in order to protect the privacy of EHR. These rules and regulations require tough measures of security while sharing and exchanging the health data. If the sharing failed to follow the rules, strong sanctioned were imposed on the violators with severe penalties. The introduction of AI and multi-agent based systems into the health data make it easy for the management to take its decisions and the actions, and ensures the communication and coordination by minimizing the errors of analysis and treatment, and by improve time required to look for the medical resources, and other medical departments. The main goal of AI-based EHR security is to create methodology, tools, and facilities for the maintenance and transfer of health data through the EHR.

Electronic Health Records are live and systems based on the patient. This makes the patient data to be accessed and handled by users who are authorized to use it. These data are in a digital format which is collected based on the already developed standards for maintaining the patients’ health records.

In this EHR, the data can be handled by the patient or an authorized doctor and the service provider. It is stored in a cloud-based servers which can be accessed only by the users (Zhang et al., 2015; Shinde & Patil, 2015). The users and the data were connected through a network. All the requests and transmissions were done through the network (Shinde & Patil, 2015). Though there were various advantages present in this EHR, it is more vulnerable to various types of attacks. This is due to its design architecture (Om & Talib, 2011). Various threats in the level of collecting data (Habib, Torjusen & Leister, 2015; Saleem, Ullah & Kwak, 2011; Chelli, 2015; Kumar & Lee, 2011; Saleem, Ullah & Yoo, 2009; Om & Talib, 2011), transmission (Ismail & Ammar, 2020; Partala et al., 2013; Niksaz, 2015; Bonab & Masdari, 2015), and storage (Santos-Pereira et al., 2013; Zhang & Liu, 2010; Drosatos et al., 2016; Fatema & Brad, 2014; Wellington, 2013) were present in this EHR’s. Due to these threats, some of the users are concerned to employ this EHR to save and transmit their health data (Ismail & Ammar, 2020). Hence, a novel methodology for providing privacy and security combining the AI-based intelligent agents and blockchain is proposed in this article.

### Contributions of the present research

- Various techniques were proposed earlier researchers for enhancing the security and electronic based health record. Enhancement of EHR systems are under process.
- A proposal of combining the AI, agent based systems along with the Blockchain based technology for providing privacy and security in the Electronic Health Record.
- The proposed AI-based DLS technology achieves better accuracy in providing security and privacy while utilizing and sharing the EHR data.

This research work is done in order to analyze the drawbacks present in the existing privacy and security preserving methodologies for the EHR data sets and to propose a novel method for providing privacy and security in it.

### Attributes of the Distributed Ledger Technology

- A DLT is a distributed digital record of transactions (Zhang, Zhong & Tian, 2017; Zhang et al., 2017). The terminology comes from its structure, where, the individual records called blocks. These blocks are linked together with each other, and in accordance with the implemented consensus protocol, in single list which is called a chain. The current implementation of DLT is now seeing in recording crypto currencies transactions. The notion of decentralization is core component; hence, any involved transactions cannot be altered without the alteration of all concurrent blocks.
- The key characteristics of the DLT include
- Decentralization
- Persistency
- Anonymity and
- Auditability
- The key advantages and features of DLT technologies, includes:
- Immutability: It means one-way writing to the ledger, hence difficult to tamper or alter a block or committed transaction.
- Irreversibility: It prevents double spending.
- Distribution of records: It means that a copy of the ledger is present with all its members.
- No Centralized Authority or third party: It is a peer-to-peer network.
- Resiliency: It is not prone to any sort of major attacks.

### Blockchain

**Figure 1:**
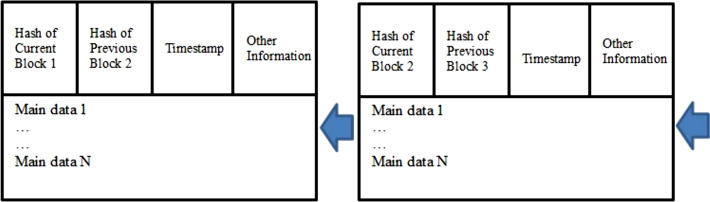
Structure of blockchain.

Blockchain (DLT) is considered to be the next big technological revolution, as it is reinventing the way we work and live (Zheng et al., 2018; Perdana et al., 2020; Sun et al., 2020). The structure of blockchain is shown in Fig. 1. The idea of the DLT was first introduced by a researcher who implemented the digital crypto currency known as Bitcoin. DLT has become an integral part of bitcoin’s operation (Li et al., 2019). For several decades, researchers have been dealing with information exchange and the transfer of money and other assets through online transactions through the Internet, where each of these transactions involved a trusted intermediary. It is provides a secure exchange and traceability in the event of any failures in case of a security breach. In a shift of paradigm, the DLT removes centralized authority which is present in-between multiple entities which are processing the financial and other transactions on data using a public ledger which is incorruptible, immutable, and decentralized in nature.

The DLT based technology can come up with acceptable results in the certification system for learning, receivable to the non-changeable and the cryptographic nature of the data based on blockchain. It can complete complex transaction operations without human intervention. The system also supports automatic execution and automatic verification. Smart contract technology can simplify the process of transaction, smart realization, making it automated and decentralized and to enhance the security of the transaction.

### AI and Multi agent based systems

**Figure 2:**
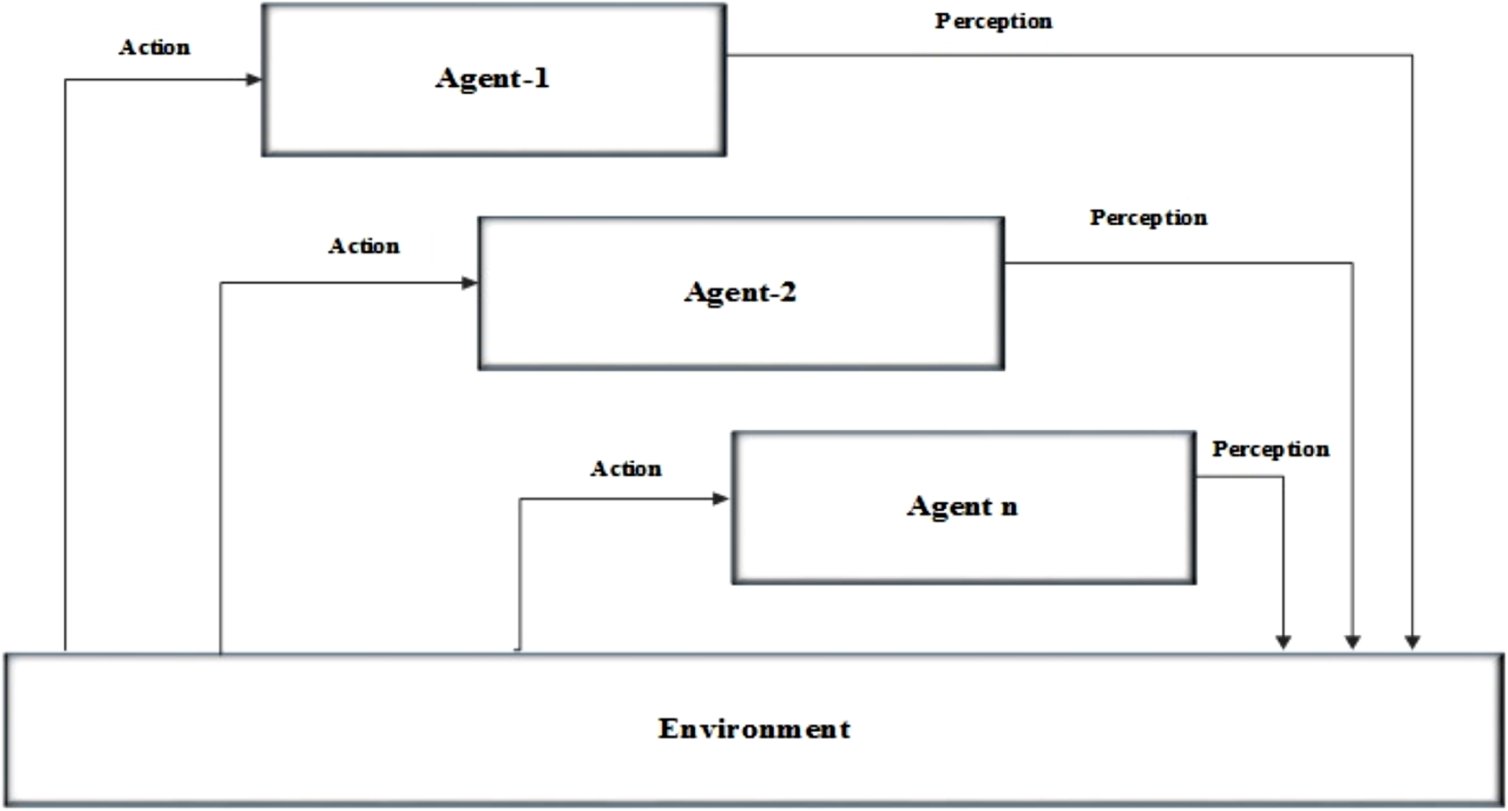
Architecture of the multi agent-based system.

AI is the process of recognizing something it has never seen before and predict the future, by extracting patterns in the past (Harel, Gal & Elovici, 2017; Bali, Garg & Bali, 2019). AI deals with the study and design of intelligent based agents which maintains the environment, takes actions which increases the chance for occurring success. Intelligent agents or adaptive & self-organizing systems are autonomous based system which is more flexible in receiving and processing the input for generating the output with respect to the input. Agent-based systems are communicating systems of distributed AI. These systems work by communicating with each other based on a set of rules and constraints in order to solve a common problem. However, agent-based systems usually consist of one learning AI agent and other Pattern of AIs. An AI-based multi-agent system is a computerized system which consists of multiple interacting intelligent agents. Multi-agent systems can solve problems which are difficult for an individual system. These intelligent based agents can get the data in the form of knowledge directly from the users. These users can be also called as environment. Intelligent agents can perform a specific task for the given inputs. These systems can process the inputs from humans or other form of agents. These agents can also be able to combine with other agents in order to perform a process. Generating a system based on the intelligent agent is not a difficult process since it can be done just by combining the existing agents with agents. This forms the multi agent based architecture. For example, a network monitoring agent can be created by combining the network system with an agent by not altering the entire mechanism. Architecture of an intelligent agent is shown in Fig. 2.

### The security and privacy opportunity

**Figure 3:**
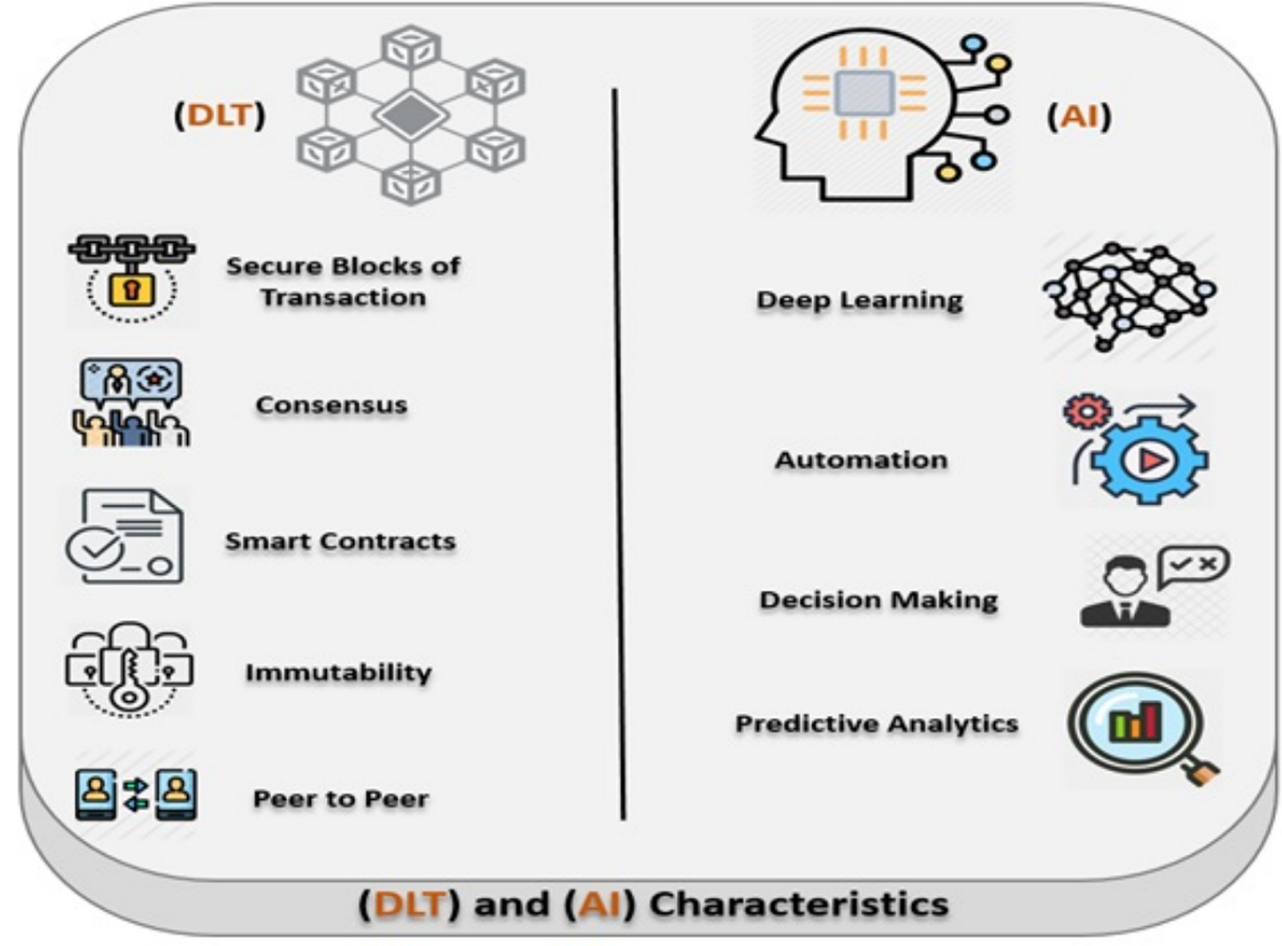
Main characteristics of the blockchain (DLT) and AI.

The DLT combined with AI integration presents a new and innovative approach to achieve smart, resilient and secure handling of EHR data. Various works has been done by previous researchers regarding the security and privacy using the Multi agent bases systems (Tsochev et al., 2018; Grzonka et al., 2018; Ahmed et al., 2020; Gruson et al., 2019; Stein et al., 2019). The fact that increased adoption of autonomous systems e.g. internet of things, opens the door for insecure and cyber-criminal activities, hence a greater need to ensure security, control and compliance. With this increased connectivity and reliance of other healthcare systems across different healthcare stakeholders (e.g., pharmacy, imagery, prescriptions, physicians, insurance) a greater need for privacy protection to keep patients and hospitals records safe and secure is critical. The gap intensifies when urgent actions are needed to protect or recover EHR transactions against cyberattacks. The utility of greater intelligence in making decisions about threats and cyberattacks can be utilized. The AI agents are used to gather the necessary threats/cyberattacks information from multiple deployed sensors to help steer the decision making process. In addition, automation of actionable policies to protect EHR can be codified into a smart contracts with will be triggered based on the given event (IF-THEN-ELSE condition statement). Figure 3 shows the main important characteristics of the Blockchain (DLT) and AI.

### Proposed methodology

**Figure 4:**
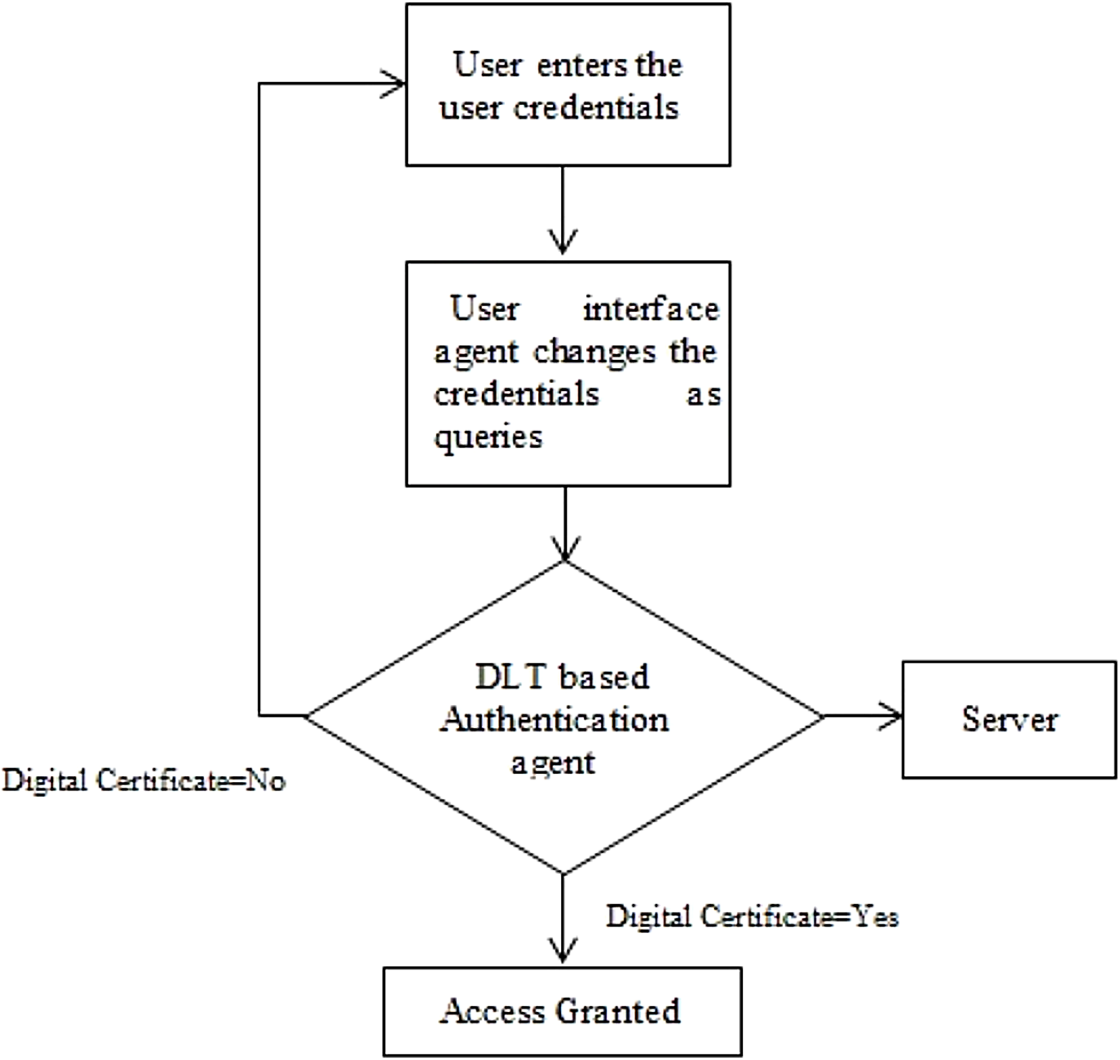
Flowchart of the proposed methodology.

The proposed methodology is designed to provide privacy and security in EHR database using the application of Artificial Intelligence and Multi Agent based Distributed Ledger System. This framework is secured since the blockchain and various software based agents are used in between the data which are available in the public servers. In this method, the user has full privilege to access the system. The user can update, edit, modify and delete the contents present in the database which is connected with the system. Architecture of the proposed system is shown in Fig. 4 which has two types intelligent agent such as the user interface agent, DLT based authentication agent. This forms the multi agent based technology. The user interface agent collects all the information about users who are accessing the database. The DLT based authentication agent generates a digital certificate for accessing or transferring the data from the server towards the connected devices. All these information’s were stored in the server. These intelligent DLT based multi agent systems provide the communication between users and the EHR service provider. Flowchart of the proposed methodology is shown in Fig. 4.

**Figure 5:**
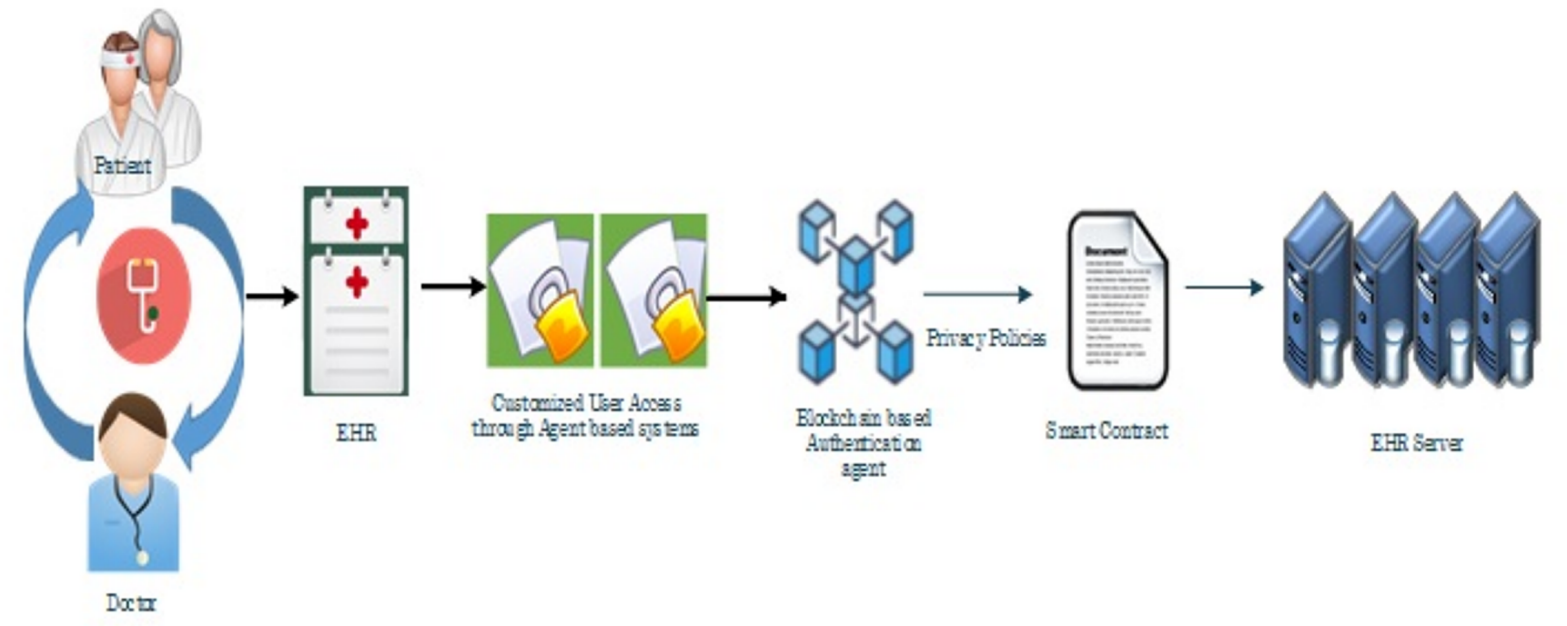
Schematic representation of the proposed methodology.

The user interface agent is also responsible for defining the protocols for defining an accessing entity as a user. These protocols are based on the already set rules. A centralized server is used to stores all the health related information of the patient. This server is connected to the authentication agent. This server also called as the health record database consists of user credentials for accessing the entire EHR system. An authentication agent is an intelligent agent which is connected with the health record database or server which can be used to validate the entry of user. It is also used to verify the user credentials present in the health database. The proposed system consists of two intelligent based connected agents such as the user interface and the DLT based authentication agent. The Authentication agent is also connected with the DLT based system which can only grant access to the users. This agent makes effective comparison of the already stored datasets of the users and the providers who are willing to access the datasets by issuing a digital certificate based on the blockchain technology. Various operations such as user interface establishment, user registration, comparison of the already existing data, and its maintenance where depicted by this intelligent based agents. The basic information such as username, passwords, user credentials, birth date, mobile number etc. and all the necessary information related to the health regarding the patient are stored in the health record database or server. Schematic representation of the proposed methodology is shown in Fig. 5.

### Working principle

**Figure 6:**
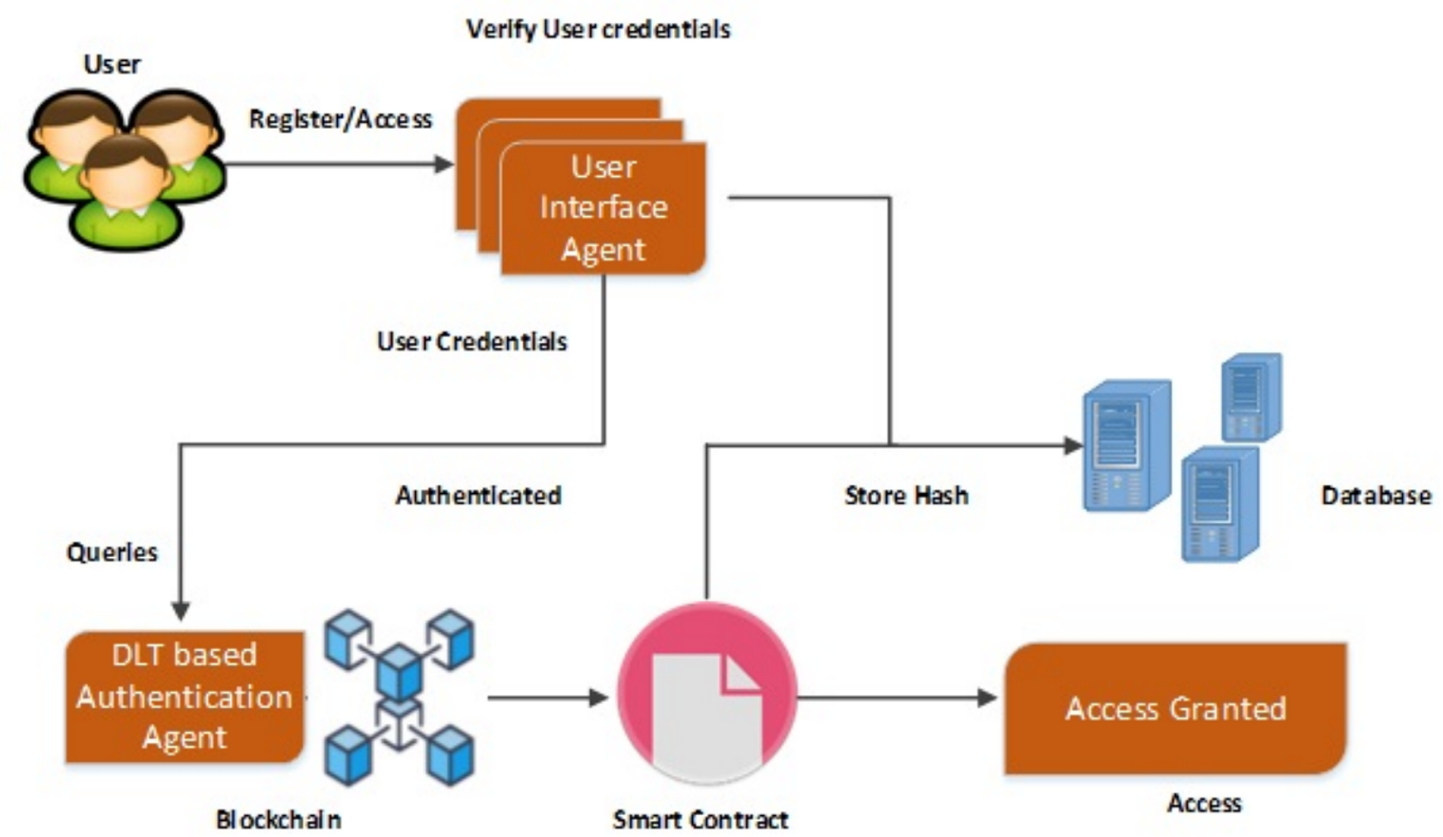
Architecture of the proposed model.

User interface agent acts as an interface between the user and the entire system. This user interface agent is used to establish the connection between the users and authentication agent. The user interface agent present in between the users and the authentication agent accept the sign in request from the user. The user can be a patient, a doctor or a provider healthcare. This user interface agent accepts the user credentials required for logging in. The user credentials can be a username and a password. The User interface agent is established through an application which can be either a website or a mobile based. The user can enter his user name and password in the provided interface. If the user forgot the username or password, it can be easily accessed by the security protocols functions present in the interface. The DLT based authentication agent receives the user name and password. All the user credentials received by the user interface agent were passed to the DLT based authentication agent for further accessing whether the credentials belongs to the saved users or not. The DLT based Authentication agent checks the obtained user name and password of the user from the stored datasets. The username and passwords were already stored in the database as shown in Fig. 6. After validating the credentials, it generates a digital certificate to the user. Connection is established in between the server and the DLT based authentication agent for accessing the EHR data. The DLT based authentication agent present in between the user interface agent and the server generates a digital certificate in order to access the data in terms of using as well as transferring. If the digital certificate is generated, then the user can access, transfer data towards the EHR server. By this method, the EHR data cannot be easily accessed by un-authorized users or by un-authorized login. Overall Architecture of the proposed model is shown in Fig. 6.

All the information regarding the logging in will be also stored in the database or the server in the form of references for next authentication process. If the credentials of the user who is willing to access the database is not stored in the server, then a user registration form will be provided so that the new user can register him for accessing the data. It is further analyzed by the authentication agent and it will grant permission to access based on the priority. Once the user is verified then he will granted permission to access the data and the connection will be established for the user to access the database.

### Algorithm for the agent based systems

Procedure for user verification

Begin

 Initialize

 for every agent V and every user U,

 Read the user credentials

 If (U = Username & password) and

 UI = <U, P>

then,

 Forward to the User Interface Agent;

else

 Enter the valid Username and Password;

 End if,

End for

Procedure for User Interface Agent

Begin

 Initialize

Each agent ${{\rm a}_{\rm i}} \in {\rm V}$

Initialize a to do list where $\left| {{{\rm l}_{\rm i}}} \right| = {\rm \; }{{\rm V}_{\rm i}}$

Check the user and its interface

 Read the user credentials

U = <UI>

then,

 Forward to the DLT based Authentication Agent;

else

 Unauthorised user;

 End if,

End for

### Algorithm for generating digital certificates

Main module of the proposed algorithm starts with a login process. A user can log in from the user interface agent with the starting time as zero. The logging in time will be 1 h where the system allows the user to obtain a digital certificate. At once the user verification is done, the user particulars were updated in the server. If the user didn’t obtained a smart contract already, then the user particulars were entered in the digital certificate with the user address stored in the DC. Then the user particulars were sent towards the execution process of smart contract. At once the user particulars were updated, the system produces a digital certificate. The algorithm for generating a digital certificate is shown below

login = Ok, Start_time == 0,

User_Verification = OK

If login == Valid and Start_time >= 1 hour:

Then: Update User_particulars;

Smart Contract = No

If login == Not Valid or Start_time <= 1 hour

Then:

DC = Digital_Certificate(User_particulars);

Address = DC_Address;

Send User_particulars to address: Excute Smart_Contract = SC_Address(User_particulars);

If Smart_Contract = Ok

Then:

Add block_credit to DC_Blockchain;

Issue (Digital_Certificate);

End If

End If

End If

### Motivation and scope

- The proposed methodology provides the health data as privacy preserved and secured.
- The proposed system is monitored and maintained by the AI-based multi agent systems.
- The proposed system consists of two intelligent based connected agents such as the user interface and the DLT based authentication agent.
- These multi agents can be used to validate the user ans also yto generate the digital certificates.
- Digital certificates are generated by blockchain based Intelligent agents for restricting the un-authorized users.
- AI and blockchain based intelligent agents communicates between the users and the EHR server through the network.

### Comparison with existing methods

In this section a detailed comparison between the proposed methodology and the existing methods for providing privacy and security for health datasets are depicted. In the CARE Model, the intelligent agent based system is employed where each and every entities and their role are monitored. In Multi Agent System for Patient-Centered method, only the intelligent agents were assigned for individual patients. In agent based medical Server and wireless sensor network, only individual agents are assigned for all the entities such as the patient, supervisor, doctor and manager. In algorithms based on biometrics based technologies, EEG, ECG and finger print sensors were employed for the authentication purpose. In the proposed method, intelligent multi agents along with the DLT based systems is used for user authentication and also for providing access to the EHR datasets. Table 1 shows the comparison of techniques for various E-health security methods.

**Table 1:** Comparison of techniques for various EHR security method.

EHR security methods	Technique used
CARE model	Modules for each actors and their role
Patient-centered multi agent system	Agents for individual users
Body sensor network and agent based medical server	Agents for patient, supervisor, doctor and manager
Biometric based method	Electrocardiogram and finger print for the authentication purpose.
Biometrics based method	Electrocardiogram and photoplethysmography for the authentication purpose.
Proposed method	Blockchain along with multi agents for user interface, authentication, smart contract for accessing the EHR data

## Conclusion and Future Work

E-Health systems can be a better alternative to maintain the medical records globally and connectedly and can be further accessed the clinical information on the basis of its requirement. Due to the fast improvement of the users among the EHR, E-Health and M-Health, applications based on mobile devices which provide medical services such as the data collections of online users were facing a challenge in securing, storing and accessing the data. Patient’s health record which was used in various treatments should be secured. These records were used by various doctors and specialists of the healthcare while providing the treatments to patients. A combination of AI bases intelligent agents and blockchain technology is proposed in this article in order to provide security and privacy for the EHR from unauthorized access and usage. Intruders can change the information, alter the entire data, introduce an unauthenticated and false data, etc. This system avoids these types of attacks caused by the un-authorized users and users with the help of intelligent agents and blockchain technology by generating a smart contract. This proposed architecture uses various AI-based intelligent based agents and blockchain for the entire process. Future enhancement in this work can be the addition of the biometric based systems for improved security.

## Supplemental Information

10.7717/peerj-cs.323/supp-1 Supplemental Information 1 Code.

## Additional Information and Declarations

### Data Availability

The following information was supplied regarding data availability: The code is available as a Supplemental File.
